# A genetic link between epigenetic repressor AS1-AS2 and a putative small subunit processome in leaf polarity establishment of *Arabidopsis*

**DOI:** 10.1242/bio.019109

**Published:** 2016-06-22

**Authors:** Yoko Matsumura, Iwai Ohbayashi, Hiro Takahashi, Shoko Kojima, Nanako Ishibashi, Sumie Keta, Ayami Nakagawa, Rika Hayashi, Julio Saéz-Vásquez, Manuel Echeverria, Munetaka Sugiyama, Kenzo Nakamura, Chiyoko Machida, Yasunori Machida

**Affiliations:** 1 Division of Biological Science, Graduate School of Science, Nagoya University, Furo-cho, Chikusa-ku, Nagoya 464-8602, Japan; 2 Botanical Gardens, Graduate School of Science, The University of Tokyo, Hakusan 3-7-1, Bunkyo-ku, Tokyo 112-0001, Japan; 3 Graduate School of Horticulture, Chiba University, 648 Matsudo, Matsudo-shi, Chiba 271-8510, Japan; 4 Graduate School of Bioscience and Biotechnology, Chubu University, 1200 Matsumoto-cho, Kasugai, Aichi 487-8501, Japan; 5 CNRS, Laboratoire Génome et Développement des Plantes, UMR 5096, Perpignan 66860, France; 6 Université de Perpignan Via Domitia, Laboratoire Génome et Développement des Plantes, UMR 5096, Perpignan F-66860, France

**Keywords:** *Arabidopsis thaliana*, AS1-AS2, Epigenetic repression, Nucleolus factors, SSU processome, Leaf polarity

## Abstract

Although the DEAD-box RNA helicase family is ubiquitous in eukaryotes, its developmental role remains unelucidated. Here, we report that cooperative action between the *Arabidopsis* nucleolar protein RH10, an ortholog of human DEAD-box RNA helicase DDX47, and the epigenetic repressor complex of ASYMMETRIC-LEAVES1 (AS1) and AS2 (AS1-AS2) is critical to repress abaxial (ventral) genes *ETT/ARF3* and *ARF4,* which leads to adaxial (dorsal) development in leaf primordia at shoot apices. Double mutations of *rh10-1* and *as2* (or *as1*) synergistically up-regulated the abaxial genes, which generated abaxialized filamentous leaves with loss of the adaxial domain. DDX47 is part of the small subunit processome (SSUP) that mediates rRNA biogenesis. In *rh10-1* we found various defects in SSUP-related events, such as: accumulation of 35S/33S rRNA precursors; reduction in the 18S/25S ratio; and nucleolar hypertrophy. Double mutants of *as2* with mutations of genes that encode other candidate SSUP-related components such as nucleolin and putative rRNA methyltransferase exhibited similar synergistic defects caused by up-regulation of *ETT/ARF3* and *ARF4*. These results suggest a tight link between putative SSUP and AS1-AS2 in repression of the abaxial-determining genes for cell fate decisions for adaxial development.

## Abstract (precis)

**Summary:** This paper reports the importance of cooperative action between the nucleus-localized epigenetic repressor and the nucleolus-localized proteins involved in ribosomal RNA processing for polarity establishment of *Arabidopsis* leaves.

## INTRODUCTION

A step of developmental events in multicellular organisms is characterized by the activation of a new genetic program and the inactivation of the previous program, which is mostly mediated by epigenetic systems. Recently, a complex of plant-specific nuclear proteins ASYMMETRIC LEAVES1 (AS1) and ASYMMETRIC LEAVES2 (AS2) (AS1-AS2) of *Arabidopsis thaliana* has been proposed to play a role in the epigenetic silencing involved in early leaf development from stem cells at the shoot apices (Phelps-Durr et al., 2005; Guo et al., 2008; Lodha et al., 2013; Iwasaki et al., 2013). Both nuclear proteins are localized as speckles, called AS2 bodies, to regions adjacent to and sometimes inside the nucleolus (Ueno et al., 2007; Luo et al., 2012a), suggesting a potential role of AS1 and AS2 in the gene silencing through nucleolus-associated events.

Leaf primordia appear around the shoot apical meristem (SAM: a group of stem cells at the shoot summit), and adaxial-abaxial (dorsal-ventral) leaf polarity is subsequently established by actions of various polarity-specific genes during the early development. The abaxialization (ventralization) is thought to proceed to adaxialization (dorsalization) because abaxial-determining genes were expressed earlier than that of adaxial-determining genes (Sawa et al., 1999; Eshed et al., 1999, 2001) and the defect of the adaxial domains results in the generation of the filamentous leaf that retains only abaxial traits (Waites and Hudson, 1995; Li et al., 2005; Ueno et al., 2007). These abaxial and adaxial genes have genetically antagonistic relationships with each other, as expression of the abaxial genes could repress that of the adaxial genes and vice versa (Bowman and Floyd, 2008; Nakata and Okada, 2013). For example, *KANADI* genes (parts of abaxial genes) directly repress *AS2* (part of adaxial genes) transcription (Wu et al., 2008).

AS1-AS2 is regarded as a key regulator for the proper confinement of the stem cell fate and the adaxial development (Waites et al., 1998; Byrne et al., 2000; Semiarti et al., 2001; Iwakawa et al., 2002, 2007; Matsumura et al., 2009). Direct repression by AS1-AS2 of the expression of at least two classes of genes is critical for leaf development, one of which is the class 1 *KNOX* homeobox genes such as *BREVIPEDICELLUS* (*BP*), *KNAT2,* and *KNAT6* (Guo et al., 2008; Ikezaki et al., 2010). AS1-AS2 interacts physically with the plant homolog of the component of Polycomb-Repressive Complex 2 and recruits this complex to these *KNOX* genes, and is involved in maintenance of levels of trimethylation of histone H3Lys27 in the *KNOX* genes (Lodha et al., 2013). The other class of direct targets is the abaxial-determining gene *ETTIN* (*ETT*)/*AUXIN RESPONSE FACTOR3* (*ARF3*) (Iwasaki et al., 2013), which is directly repressed by the binding of AS1-AS2 to its promoter region. In addition, AS1-AS2 also indirectly represses expression of *ETT*/*ARF3* and functionally redundant gene *ARF4* through activating the miR390-tasiR-ARF pathway, which is essential for the adaxial development. AS1-AS2 is required for maintaining levels of CG methylation in the *ETT*/*ARF3* coding region (Iwasaki et al., 2013); thus AS1-AS2 plays a key role in the antagonistic action by adaxial/abaxial factors and in epigenetic repression of *ETT*/*ARF3*.

Many factors are involved in leaf adaxialization in an AS1-AS2 dependent manner, in the *as1* or *as2* mutation background various modifier mutations have been identified that markedly enhance the defects of adaxial development to generate abaxialized filamentous leaves with loss of the adaxial domain. Causative mutations occur in genes that are involved in the biogenesis of small RNAs, chromatin modification and cell cycle progression (Williams et al., 2005; Machida et al., 2015)*.* Mutations or disruptions of some ribosomal protein genes (Pinon et al., 2008; Yao et al., 2008; Horiguchi et al., 2011; Szakonyi and Byrne, 2011) and genes for ribosome biogenesis (Ueno et al., 2007; Huang et al., 2014) also act as enhancers of the *as1* and *as2* phenotypes. Collectively, many modifiers are weak alleles of the essential genes and strongly enhance the adaxial defects of *as1* and *as2.* These findings suggest that AS1-AS2 and an enhancer protein act cooperatively on adaxial development through the epigenetic repression of *ETT*/*ARF3* and *ARF4* genes, however mechanisms of these cooperative actions are still unknown.

In the present study, we identified a new enhancer mutation in the *Arabidopsis RH10* gene which potentially encodes an ortholog of yeast Rrp3 and human DDX47 that belong to the DEAD-box RNA helicase family. This helicase family has an indispensable role in gene regulation through RNA metabolism and many of them locate in the nucleolus. Rrp3 and DDX47 are parts of the nucleolar protein complex designated as the small subunit (SSU) processome that is a large ribonucleoprotein complex involved in 18S rRNA biogenesis, assembling the SSU of ribosomes (Dragon et al., 2002; Granneman et al., 2006; Phipps et al., 2011), that is ubiquitous in eukaryotes (Feng et al., 2013). Although DDX47 is necessary for maintaining the pluripotency of mouse stem cells (You et al., 2015), the subfamily of this helicase has yet to be intensively studied in relation to the development of multi-cellular organisms. Here, we report that RH10 represses cooperatively with AS1-AS2, the expression of abaxial-determining genes to promote the adaxial development of leaves. We also show that two other components that are related with the putative *Arabidopsis* SSU processome have similar functions in leaf development. Taken together with earlier results, the present results reveal a genetic link between AS1-AS2 and a protein complex such as the SSU processome for the polarity establishment of leaves.

## RESULTS

### A novel enhancer mutation of *as2* that causes adaxial defects of leaves is present in the gene for DEAD-box-type RNA helicase RH10

We found the strong enhancer mutant in our library of mutagenized seeds of *as2-1* (Kojima et al., 2011; Ishibashi et al., 2012), which showed filamentous and trumpet-like leaves and was named *enhancer of asymmetric leaves two* (*east2*). Molecular and genetic analyses showed that *east2* was a recessive single mutation in the 9th exon in At5g60990, which is predicted to encode a protein belonging to the DEAD-box RNA helicase family called RNA HELICASE10 (RH10) (Fig. S1A-D) (Aubourg et al., 1999). In *east2*, the cytosine residue at nucleotide position 821 was converted to a thymine residue to replace the threonine residue at position 273, near motif IV, with an isoleucine residue (T273I). There are no *RH10* knockout line and point mutants in public collections. In *Arabidopsis*, the amino acid sequence of RH10 showed the highest identity with that of RH36 (42.4%). RH10 had high amino acid identities with rice RH10 (63.0%), human DDX47 (60.5%), mouse DDX47 (59.8%), and budding yeast Rrp3 (50.1%) (Fig. S1D). Transcripts of *RH10* were detected in all tissues and organs examined (Fig. S1E). The phenotype of *east2* (see below) was complemented by the recombinant *RH10* cDNA (Fig. S1F). Accordingly, this mutation was designated as *rh10-1*.

**Fig. 1.**
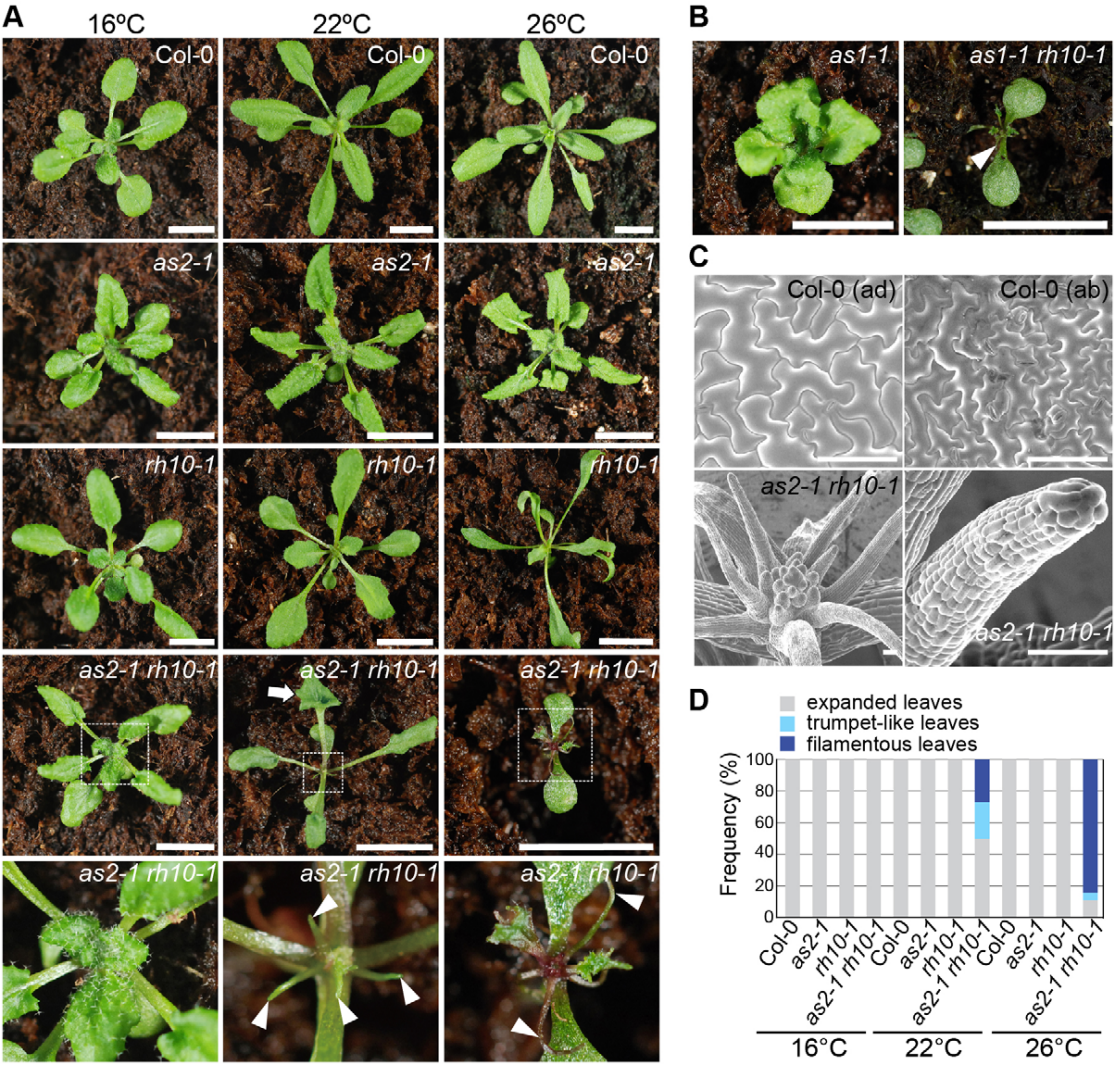
**The *rh10-1* mutation enhanced leaf-phenotypes in *as* mutants.** (A) Gross morphologies of Col-0 and indicated mutants. Indicated plants were grown on soil at 16°C, 22°C, and 26°C. In the bottom row, magnified images of shoot apices of the corresponding to *as2-1 rh10-1* of the previous row (white-dotted boxes) are shown. (B) Gross morphologies of indicated mutants*.* (C) Magnified views observed by SEM of the epidermal cells and the shoot apex of indicated plants grown at 26°C. ad, adaxial side; ab, abaxial side. (D) Quantitative analysis of leaf polarity in plants grown under different temperature conditions. Rosette leaves were classified as expanded, trumpet-like (white arrow in A), and filamentous (white arrowheads in A and B) leaves. Frequency is defined as the ratio of the number of plants with more than one trumpet-like or filamentous leaf to the total number of plants examined. Plants that generated both trumpet-like and filamentous leaves were included in those generating filamentous leaves. Plants (*n=*17-163) were used for each count. 21-day-old plants were used in (A-D). Scale bars=10 mm in A,B, 100 μm in C.

The *rh10-1* phenotype was indistinguishable from that of wild type at 16°C (Fig. 1A), however it generated a weak phenotype of pointed leaves at 22°C which became narrower at 26°C (Fig. 1A), suggestive of a temperature-sensitive mutation in *rh10-1*. The level of the *rh10* transcript in the mutant was similar to that of the *RH10* transcript in Col-0 (Fig. S1E), which suggests that the temperature-sensitive mutation might be due to decrease in its enzymatic activity and/or protein amounts.

Although the *as2-1 rh10-1* double mutant at 16°C produced leaves that were indistinguishable from those of the *as2-1* single mutant (Fig. 1A), at 22°C approximately 50% of the leaves produced were short, filamentous, and trumpet-like (Fig. 1A,D). The defectiveness of *as2-1 rh10-1* leaves was enhanced at 26°C as 90% of their rosette leaves were filamentous (Fig. 1A,D). Shapes of epidermal cells of the filamentous leaves were simple and rectangular, similar to those of a petiole, but different from those of flat leaves of wild-type plants (Fig. 1C). These results suggest that the adaxial-abaxial leaf polarity was more severely disrupted in *as2-1 rh10-1* at 26°C, and similar abnormalities of leaf phenotypes were observed in *as1-1 rh10-1* (Fig. 1B).

**Fig. 2.**
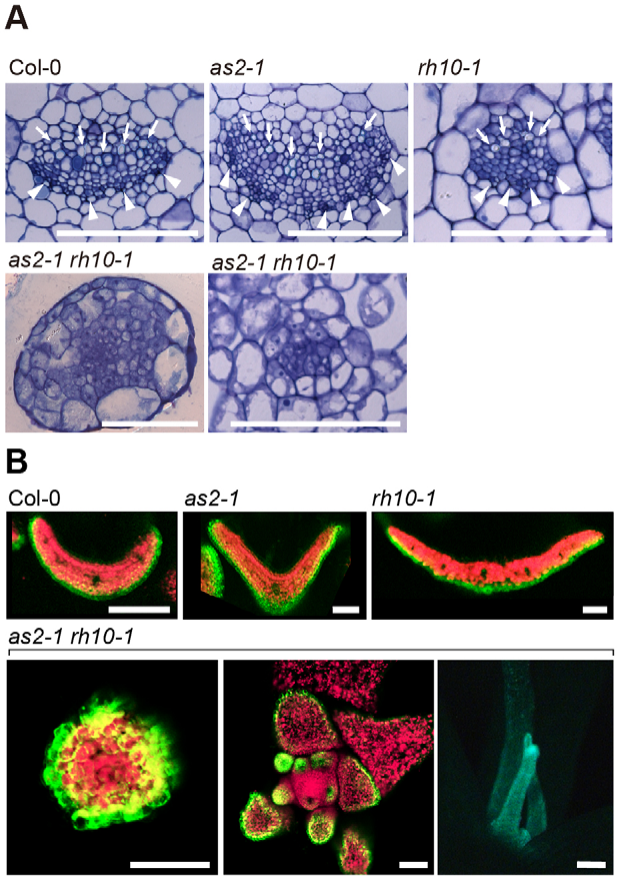
**The vascular patterns and expression patterns of *FILp*:*GFP* in the rosette leaves of *as2 rh10.*** (A) Histology of transverse sections of leaves of indicated plants grown at 26°C. Arrows and arrowheads show xylem and phloem cells, respectively. Sections of filamentous leaves without vascular tissues (left) and with disorganized vascular tissues (right) of *as2-1 rh10-1* are shown. (B) Top row: fluorescence signals of *FILp*:*GFP* in transverse sections of leaf primordia of indicated plants grown at 26°C. Bottom row: fluorescence signals of *FILp*:*GFP* in transverse sections of the filamentous leaf of *as2-1 rh10-1* grown at 26°C (left). The external views of the shoot apex (middle) and the filamentous leaf (right) are shown*.* Green, signals due to GFP; red, autofluorescence. Scale bars=100 μm.

We observed the vascular patterns and GFP signals due to FILp:GFP (FIL promoter-fused gene for green fluorescent protein, Watanabe and Okada, 2003) as an abaxial marker. In the leaves of either single mutant grown at 26°C, the xylems were located on the adaxial sides and the phloems were on the abaxial sides, similar to those observed in the wild type (Fig. 2A). The filamentous leaves of *as2-1 rh10-1* at 26°C showed primitive or no vascular tissue without apparent xylem cells inside the bundle sheath (Fig. 2A), suggesting defects in differentiation of xylem cells on the adaxial side. As shown in Fig. 2B, the GFP signals from FILp:GFP were detected on the abaxial side of the leaves of wild type, *as2-1*, and *rh10-1* plants, and on the surface of the filamentous leaves and the entire young primordia of *as2-1 rh10-1.* These results show that *as2-1 rh10-1* was defective in development of the adaxial domain of leaves, which generated abaxialized leaves. Apparently, there is a tight genetic interaction between the *AS2-AS1* and *RH10* for establishing leaf polarity.

### RH10 is localized to the nucleolus and involved in processing pre-rRNAs

**Fig. 3.**
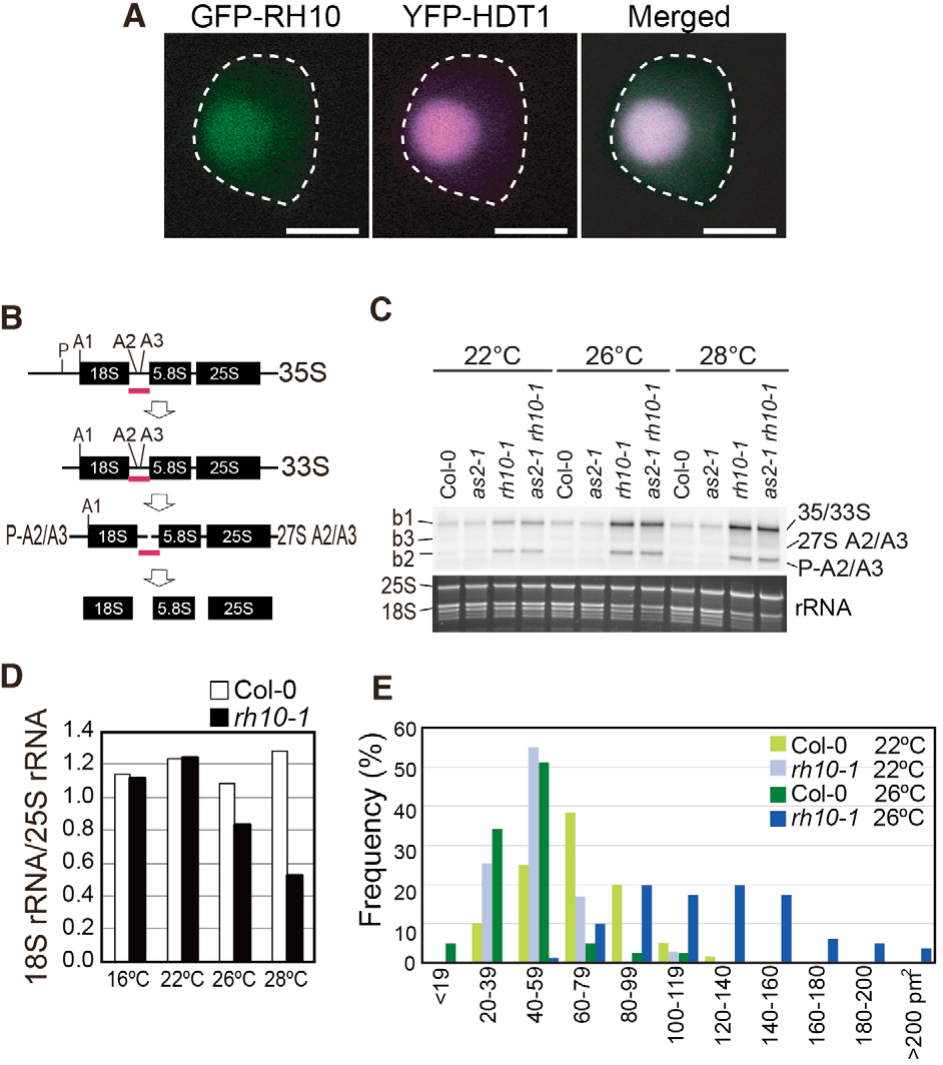
***RH10* is involved in the pre-rRNA processing in nucleolus.** (A) Localization of GFP-RH10 to the nucleolus of leaf primordial cells of *Arabidopsis*. Transgenic plants expressing *GFP-RH10* and *HDT1-YFP* (used as a marker for the nucleolus) were grown on MS plates at 22°C. The profile of the nucleus is indicated by a dashed line. Scale bars=3 μm. (B) Proposed scheme of pre-rRNA processing in *Arabidopsis* (Zakrzewska-Placzek et al*.*, 2010). Pink bars indicate the positions of a probe for northern blot analysis to detect pre-rRNAs. P, A1, A2, and A3 represent earlier processing sites around the 18S rRNA. (C) Accumulation of pre-rRNAs in the *rh10-1* mutant. Plants were grown at 22°C, 26°C, and 28°C. Total RNAs were prepared from the shoot apices of 15-day-old plants. Pre-rRNAs were detected by northern blot analysis using the RNA probe indicated in panel B. Three bands denoted by b1, b2 and b3 that may correspond with pre-rRNAs are indicated (see text). 25S and 18S rRNA bands visualized by staining with GelRed are shown as an equal-loading control. (D) Relative values of the 18S/25S rRNA ratio in *rh10-1* and the 18S/25S rRNA ratio in Col-0*.* (E) Distribution patterns of the nucleolus size in Col-0 and *rh10-1* grown at 22°C and 26°C. Area sizes of nucleoli (*n*=88-295) in two plants were measured for each of the four experiments listed in this graph and numbers of nucleoli that were classified into eleven groups indicated below the graph were counted. Area sizes of *35S:Nucleolin:GFP* fluorescence in cells of shoot apices were measured with ImageJ software.

We constructed the functional *GFP:RH10* chimeric gene (Fig. S1F), and examined the sub-cellular localization of signals due to GFP in *Arabidopsis*. We used the YFP:HDT1 chimeric protein as a nucleolus marker (Ueno et al., 2007). The fluorescent signal of GFP:RH10 was colocalized with that of YFP-HDT1 in the nucleolus (Fig. 3A), suggesting the nucleolar location of RH10.

We examined the effect of the *rh10* mutation on levels of precursors of ribosomal RNAs (pre-rRNAs) by northern blot with RNA isolated from shoot apices of plants grown at 22°C, 26°C, and 28°C, and a probe specific for the internal transcribed spacer1 (ITS1) sequence (colored red in Fig. 3B). As depicted in Fig. 3B, in *Arabidopsis* (Zakrzewska-Placzek et al., 2010), the 35S pre-rRNA corresponding to the primary transcript is rapidly cleaved at a specific site, named the P site (Saéz-Vasquez et al., 2004), in the 5′-external transcribed spacer (5′ETS) to generate 33S pre-rRNA, which is cleaved at either A2 or A3 sites in ITS1 to generate P-A2, P-A3 and 27S A2/A3 pre-rRNAs. Here we designated the pre-rRNA as P-A2/A3 because it was difficult to discriminate P-A2 from P-A3 by the experiment in the present study. These precursors undergo several processing steps to eventually generate mature 18S, 5.8S, and 25S rRNAs. The 35S and/or 33S (35/33S), P-A2/A3 and 27S A2/A3 pre-rRNAs could be detected with the probe.

As shown in Fig. 3C, three bands (denoted by b1, b2, and b3) that corresponded to these pre-rRNA molecules were detected. These precursors were detected as faint bands in wild-type (Col-0) and *as2-1* plants grown at all the temperatures. The signals of b1 corresponding to 35/33S pre-rRNAs potentially containing the primary transcript and the cleavage product at the P site, and those of b2 corresponding to P-A2/A3, were higher in the *rh10-1* and *as2-1 rh10-1* than those in wild-type and *as2-1* plants even at 22°C. Signal intensities of b1 and b2 in *rh10-1* and *as2-1 rh10-1* were markedly increased at 26°C and 28°C. Since b1 and b2 signals were also detected with the probe between the P site and the A1 site (data not shown), b1 and b2 bands contain the 5′ETS region, and b2 corresponds to the P-A2/A3 pre-rRNA, which is similar in terms of size to the 23S/22S pre-rRNA of yeast (O'Day et al., 1996). Signals of b3 corresponding to the 27S A2/A3 pre-rRNA were slightly increased at higher temperatures. These data showed that at least 33S and P-A2/A3 pre-rRNAs were significantly accumulated in *rh10-1* and *as2 rh10-1* at high temperatures.

These results suggest that the *rh10* mutation mainly prevents 35S/33S and P-A2/A3 pre-rRNAs from being processed or degraded, RH10 might be involved in early stages of the processing reactions of 35/33S and P-A2/A3 pre-rRNAs. Although the accumulation of the 35S/33S pre-rRNA might be explained by up-regulation of rDNA transcription, the reduction of the ratio of 18S rRNA/25S rRNA as mentioned in the next section supports the processing impediment in *rh10-1*. These results are consistent with those obtained by experiments with yeast Rrp3 and human DDX47 showing that they are involved at least in cleavages at the A1 and/or A2 sites during early stages of processing (O'Day et al., 1996; Granneman et al., 2006; Sekiguchi et al., 2006).

### The ratio of 18S rRNA/25S rRNA was reduced and nucleoli were enlarged in the *rh10-1* mutant at high temperatures

We examined by quantitative one-step RT-PCR alteration of the ratios of 18S to 25S rRNAs in wild-type and *rh10-1* plants grown at various temperatures. The ratio was reduced in *rh10-1* under higher temperature conditions (Fig. 3D: 79% at 26°C and 46% at 28°C as compared to each value in the wild type). Considering the roughly constant levels of the 25S rRNAs in gel (Fig. 3C), the imbalanced ratio of 18S rRNA/25S rRNA in *rh10-1* at higher temperatures might have been caused by the reduction of 18S rRNA.

We examined size differences of nucleoli in *rh10-1* mutant cells. To this end we observed fluorescent signals from NUC1-fused GFP protein, as a nucleolar marker, in the young leaves of wild-type and *rh10-1* plants grown at 22°C and 26°C. Fluorescence signals due to the NUC1-GFP were expanded in the *rh10-1* mutant grown at 26°C as compared with those in Col-0 and *rh10-1* plants grown at 22°C (Fig. 3E), which suggests that the mutation in *RH10* causes increases in the size of nucleoli. Two genes *DOMINO* and *AtREN1*, mutations of which affect the nucleolar size, have been reported (Lahmy et al., 2004; Reňák et al., 2014); however, levels of transcripts of these genes were not affected by *rh10-1* (data not shown).

### Transcript levels of abaxial genes and class 1 *KNOX* genes were elevated in *as2 rh10* at high temperatures leading to the adaxial defect

We conducted microarray analysis (with the Affymetrix GeneChip) of mRNAs from shoot apices of Col-0, *as2-1, rh10*, and *as2-1 rh10* grown at 26°C to obtain gene expression profiles in the tissues (Dataset-RH10-26). We performed cluster analysis of Dataset-RH10-26 by the knowledge-based fuzzy adaptive resonance theory (KB-FuzzyART) using Gene-list-4 (Takahashi et al., 2008, 2013) (Table S1). Constructed clusters are shown in Table S2 and Fig. S2A.

Since AS1-AS2 acts as a transcriptional repressor of *BP*/*KNAT1* and *ARF3*/*ETT* genes (Guo et al., 2008; Iwasaki et al., 2013), we focused on clusters including genes whose transcript levels were increased in the a*s2-1 rh10-1* double mutant as compared with those in the wild type. Clusters h1, h4, h6, h14, h15 and h17 included such up-regulated genes (Table S2; Fig. S2A). These clusters had *ETT*/*ARF3*, *ARF4, KAN2*, *YAB5, BP/KNAT1, KNAT2*, *KNAT6*, *STM* (Table S3), which have also been identified downstream of *EAL* and *ELO3* (Takahashi et al., 2013). We also applied KB-FuzzyART to cluster analysis of gene expression profiles obtained at 22°C (Dataset-RH10-22) (Table S4; Fig. S2B). The abaxial-determining genes and class 1 *KNOX* genes described above, except *YAB5*, were not up-regulated in *as2-1 rh10-1* at 22°C (Table S5). Increased transcript levels of genes, including abaxial genes such as *ETT/ARF3*, *ARF4* and *KAN2*, at 26°C seem to have a correlation with the phenotypes of abaxialized filamentous leaves of *as2-1 rh10-1* at 26°C.

**Fig. 4.**
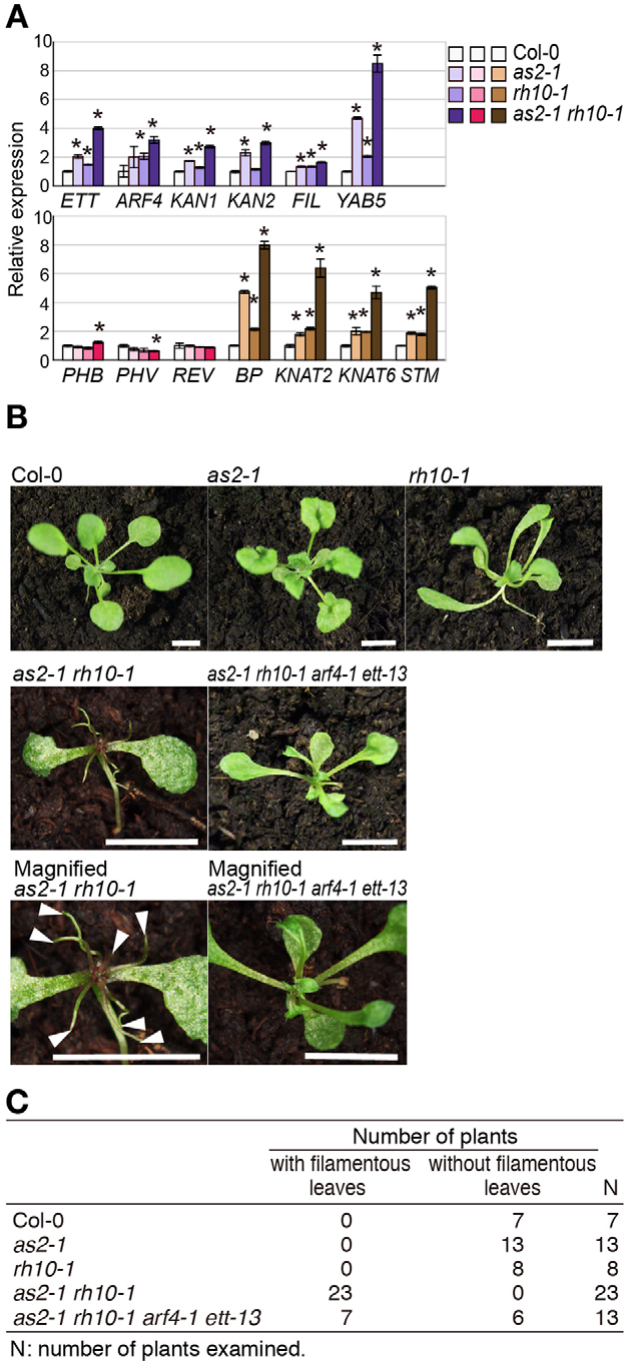
**Transcript levels of genes involved in the determination of leaf polarity and class 1 *KNOX* genes, and effects of *ett-13* and *arf4-1* on *as2-1 rh10-1* phenotypes.** (A) Levels of transcripts in the shoot apices of Col-0 and mutants. Plants were grown at 26°C. Total RNA was prepared from shoot apices of 15-day-old plants, and transcript levels were examined by quantitative real-time RT-PCR. Each value was normalized by reference to the level of *EF1* transcripts. The values from Col-0 plants were set arbitrarily at 1.0. Bars indicate the s.d. among more than three biological replicates. White-blue-coloured graphs: abaxial genes; white-pink-coloured graphs: adaxial genes; white-brown-coloured graphs: class 1 *KNOX* genes. Significant differences from wild type were evaluated by Student's *t*-test and are represented by asterisks (*P<0.01). (B) Suppression of leaf phenotypes of *as2-1 rh10-1* by *ett-13 arf4-1*. Wild-type and mutant plants with indicated mutations were grown on the soil at 26°C for 23 days. Magnified images of shoot apices of *as2-1 rh10-1* and *as2-1 rh10-1 ett-13 arf4-1* in the bottom panel were taken as different photographs of the corresponding plants in the middle panel. Arrowheads indicate filamentous leaves. Scale bars=10 mm. (C) Number of plants that generated filamentous leaves or did not. Wild-type and mutant plants with indicated mutations were grown on the soil at 26°C for 23 days.

We confirmed the transcript levels of these genes in the shoot apices of plants grown at 26°C by quantitative real-time RT-PCR (Fig. 4A; Table S6). The accumulated transcript levels of the genes involved in the abaxialization, *ETT*/*ARF3*, *ARF4*, *KAN1*, *KAN2*, and *YAB5*, were increased by two- to eight-fold in *as2-1 rh10-1* as compared with those in wild-type plants (Col-0) (Fig. 4A). *ETT/ARF3* and *ARF4* transcript levels were increased by two-fold in each of *rh10-1* and *as2-1* single mutants compared with those levels in wild-type plants. Transcript levels of the class 1 *KNOX* genes *BP*, *KNAT2*, *KNAT6*, and *STM* were markedly increased (by five- to eight-fold) in *as2-1 rh10-1*, compared with two- to four-fold increases in each single mutant. Transcript levels of adaxial genes *HD-ZIP III* (*PHB*, *PHV*, and *REV*) in the mutants did not significantly differ from those in the wild-type plants. These results showed that the *as2-1 rh10-1* double mutation causes a synergistic enhancement of transcript levels of the abaxial genes and class 1 *KNOX* genes in shoot apices at 26°C.

We also examined transcript levels of genes for nucleolar proteins such as two NUCLEOLINs, AtFIBRILLARIN, TITAN, DOMINO and AtREN1 in *as2-1 rh10-1*, but synergistic enhancement of transcript levels was not observed (data not shown).

Elevated expression levels of these *KNOX* genes do not contribute to adaxial-abaxial polarity development in *as1* and *as2* leaves (Ikezaki et al., 2010). By introducing *ett-13* and *arf4-1* mutations into the *as2-1 rh10-1* mutant, we examined effects of increased levels of *ETT/ARF3* and *ARF4* transcripts on the defects in the adaxial development in *as2-1 rh10-1.* Although 90% of the *as2-1 rh10-1* double mutant plants grown at 26°C produced filamentous leaves (Fig. 1D), half of the *as2-1 rh10-1 ett-13 arf4-1* quadruple mutants at 26°C generated no filamentous leaves, and some generated trumpet-like leaves plus narrow and expanded leaves with the adaxial-abaxial polarity (Fig. 4B,C). Thus the *ett-13* and *arf4-1* mutations significantly suppressed the adaxialization defect of *as2-1 rh10-1*, suggesting that up-regulation of *ETT/ARF3* and *ARF4* in *as2-1 rh10-1* causes the severe defect in adaxialization at 26°C, at least in part.

### Mutations of *NUC1* gene encoding nucleolin involved in pre-rRNA processing affect leaf polarity establishment in *as1* and *as2*

In the previous section we showed that RH10 is a homolog of human DDX47 and yeast Rrp3 (Fig. S1D; Fig. 3C); Rrp3 and DDX47 are reportedly parts of the SSU processome (Granneman et al., 2006; Phipps et al., 2011; You et al., 2015). Using information reported by Phipps et al. (2011), we conducted *in silico* analyses based on the similarity of protein sequences to detect orthologous proteins of the putative SSU processome in *Arabidopsis*. As shown in Table 1 (and Table S7), 74 protein sequences of *Arabidopsis*, corresponding to 54, including RH10 and two nucleolins (NUC1 and NUC2), out of the 72 proteins in yeast, were identified by these analyses. These results predict the presence of a SSU processome-like complex in *Arabidopsis*.

**Table 1.**
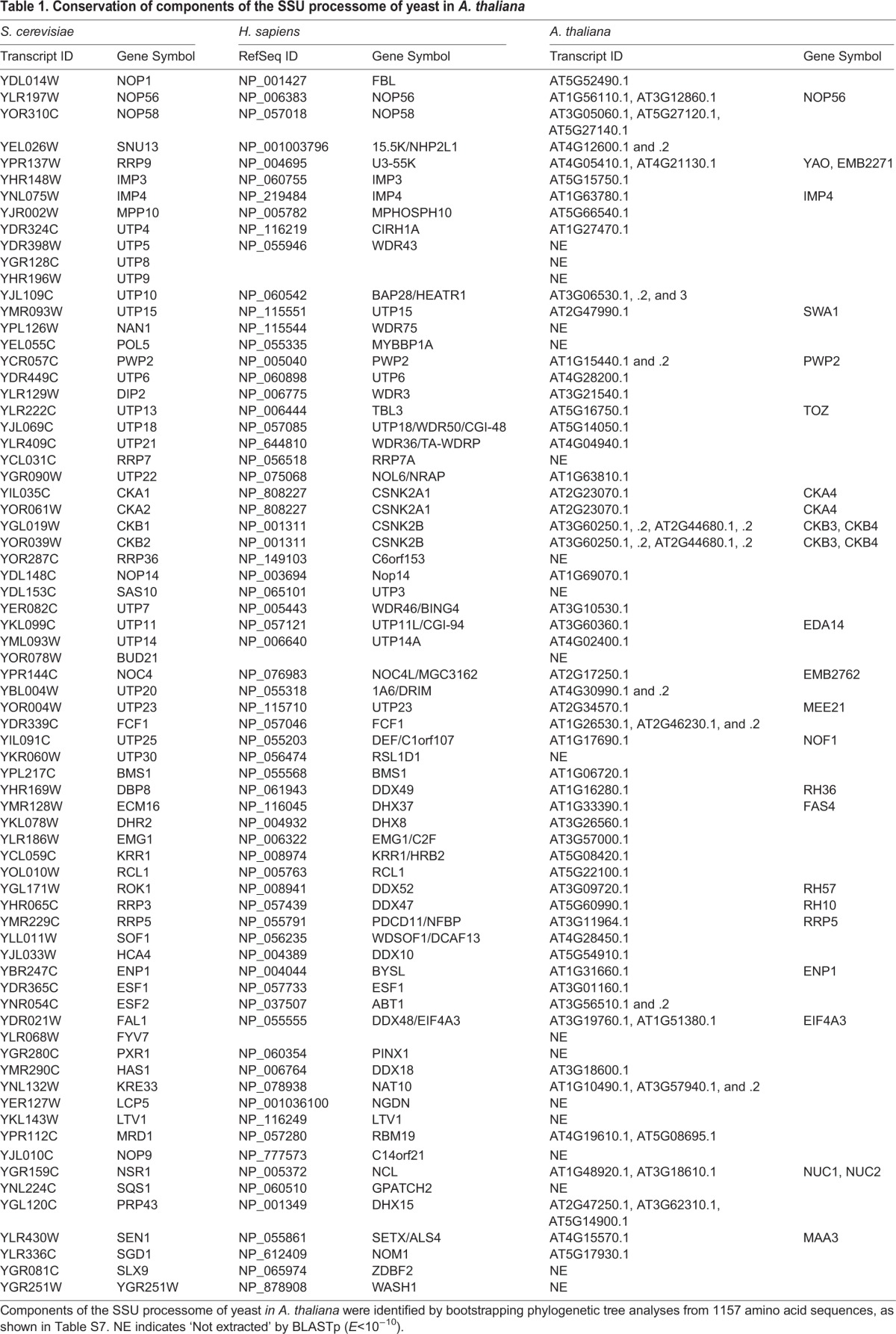
**Conservation of components of the SSU processome of yeast in *A. thaliana***

**Fig. 5.**
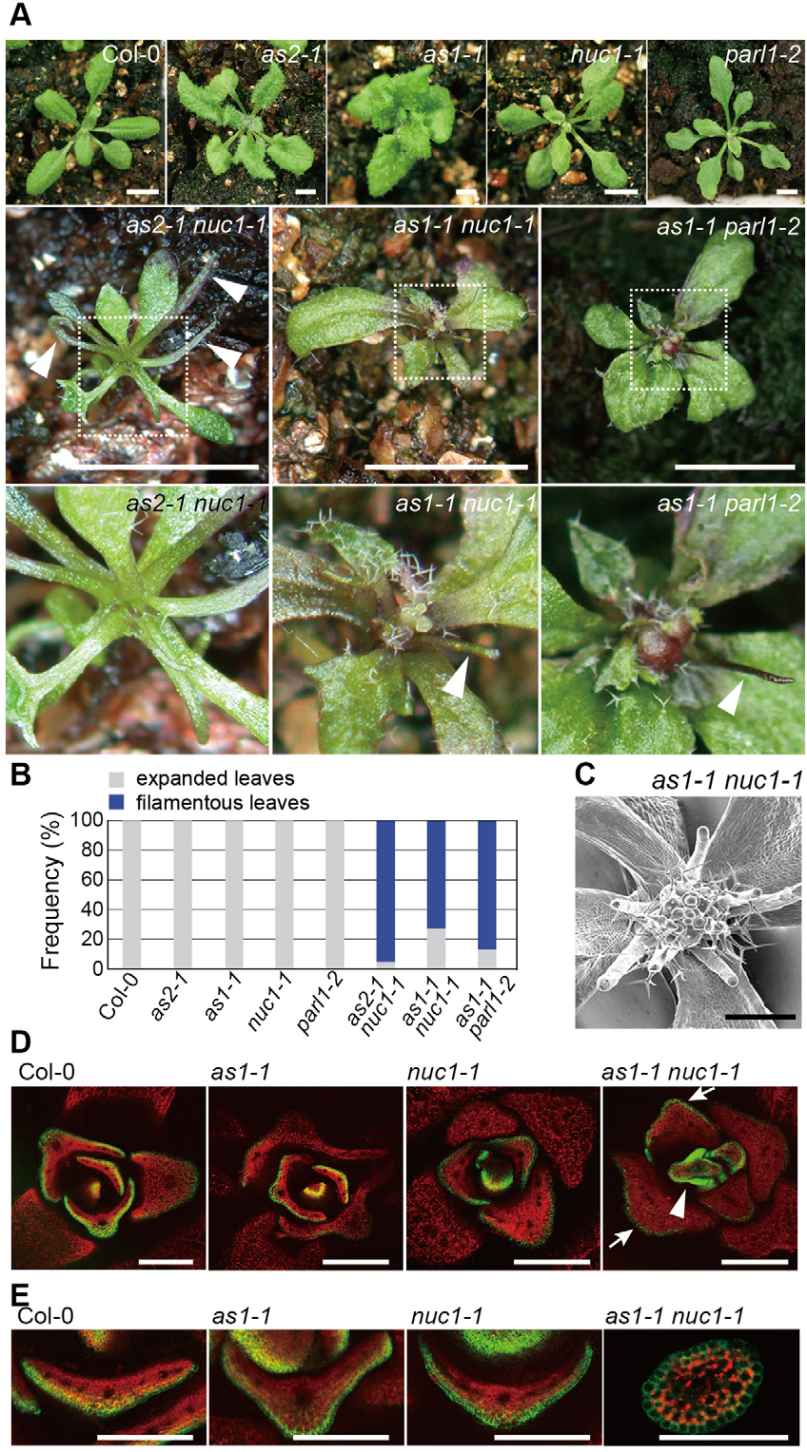
**The *nuc1* mutation enhanced leaf-phenotypes in *as2-1* and *as1-1* mutants.** (A) Gross morphologies of indicated plants. Indicated plants were grown on the soil at 22°C and photographed at 25 days. White arrowheads indicate filamentous leaves. Magnified views of the shoot apices in *as2-1 nuc1-1*, *as1-1 nuc1-1*, and *as1-1 parl1-2* plants, which correspond to the white-dotted boxed regions of each respective panel, are shown. (B) Quantitative analysis of leaf polarity in the wild-type and mutant plants. Rosette leaves classified into filamentous and expanded leaves were counted on each plant at 22 days. Plants (*n*=38-106) were used for each count. Frequencies of filamentous leaf were determined as described in Fig. 1D. (C) Magnified view of the shoot apex of an *as1-1 nuc1-1* plant observed by SEM at 28 days. (D,E) Expression patterns of *FILp*:*GFP* in leaf primordia of Col-0 and respective mutant plants. Fluorescence signals are shown in a transverse section of leaf primordia of indicated plants, and of a filamentous leaf of *as1-1 nuc1-1* grown on the soil at 22°C for 25 days. The view of filamentous leaves in a 28-day-old *as1-1 nuc1-1* plant is shown (the bottom row, right)*.* Arrows indicate GFP signals in abaxial epidermis; the arrowhead indicates GFP signals over entire epidermis. Green, GFP signals; red, autofluorescence. Scale bars*=*5 mm in (A); 500 μm in (C); 200 mm in (D); 100 mm in (E).

The prediction allowed us to examine the mutation in *NUC1* for their involvement in leaf polarity determination since nucleolin is a major protein in the nucleolus that is involved in the processing of pre-rRNAs at various stages and the single mutant exhibit a pointed narrow shape of leaves, which is observed often in other modifier mutations including *rh10* (Fig. 5) (Pontvianne et al., 2007; Kojima et al., 2007; Petricka and Nelson, 2007). In addition, human nucleolin is reported to be an assembly intermediate of the SSU processome and its candidate components (Turner et al., 2009; Phipps et al., 2011).

To investigate genetic interactions between mutations (*nuc1-1* and *parl1-2*) in the *NUC1* gene and *as2* (or *as1*), we generated the *as2-1 nuc1-1*, *as1-1 nuc1-1*, and *as1-1 parl1* double mutants (Fig. 5). The *as2-1 nuc1-1* double mutant showed severe defects in leaf shape and filamentous leaves were efficiently formed, although a few normally shaped leaves and markedly narrow leaves were also generated (Fig. 5A). Filamentous leaves were not observed in *nuc1-1* or *parl1-2* single mutants (Fig. 5A). Frequencies of filamentous leaves in *as2-1 nuc1-1*, *as1-1 nuc1-1* and *as1-1 parl1-2* double mutants were 95% (*n=*82), 73% (*n=*91) and 87% (*n*=45), respectively (Fig. 5B)*.* These results suggest that *NUC1* is required together with *AS1* and *AS2* for the formation of flat and symmetric leaves. Analyses of transcripts in a*s1-1 nuc1-1* showed that abaxial genes (*ETT/ARF3*, *FIL*, *YABY5*, *KAN1*, and *KAN2*) and all four class 1 *KNOX* genes were increased by five- to sixteen-fold (Fig. S3A). The mutation phenotype of filamentous leaves was suppressed by introduction of the *ett-13* mutation into *as2-1 nuc1-1* (data not shown). The high efficiency of filamentous leaf generation and patterns of the up-regulation of downstream genes in *as1-1 nuc1-1* are similar to those in *as2-1 rh10-1* and *as1-1 rh10-1* (Figs 1 and 4).

We observed expression patterns of the *FILp:GFP* fusion gene in leaf domains of wild type (Col-0), *as1-1*, *nuc1-1*, and *as1-1 nuc1* (Fig. 5D,E). Filamentous leaves generated in *as1-1 nucl-1* exhibited GFP signals over their entire epidermis, suggesting that the leaves are abaxialized, similarly as seen in *as2-1 rh10-1*.

### Mutation of the *RID2* gene encoding nucleolar rRNA methyltransferase involved in pre-rRNA processing affect leaf polarity establishment in *as2*

We also examined the effect of a mutation in the *ROOT-INITIATION-DEFECTIVE2* (*RID2*) gene (Ohbayashi et al., 2011) on the adaxial-abaxial organization of leaves on the *as2* background. *RID2* encodes an orthologous protein of yeast Bud23, which is nucleolus-localized rRNA methyltransferase that exhibits tight functional and physical interactions with some of the SSU processome components (Sardana et al., 2013, 2014; Zhu et al., 2016). *RID2* encodes a nucleolar protein that is involved in the processing of pre-rRNAs at various stages from early steps, and its single mutant also shows a typical pointed shape of leaves like other modifier mutants (Ohbayashi et al., 2011).

**Fig. 6.**
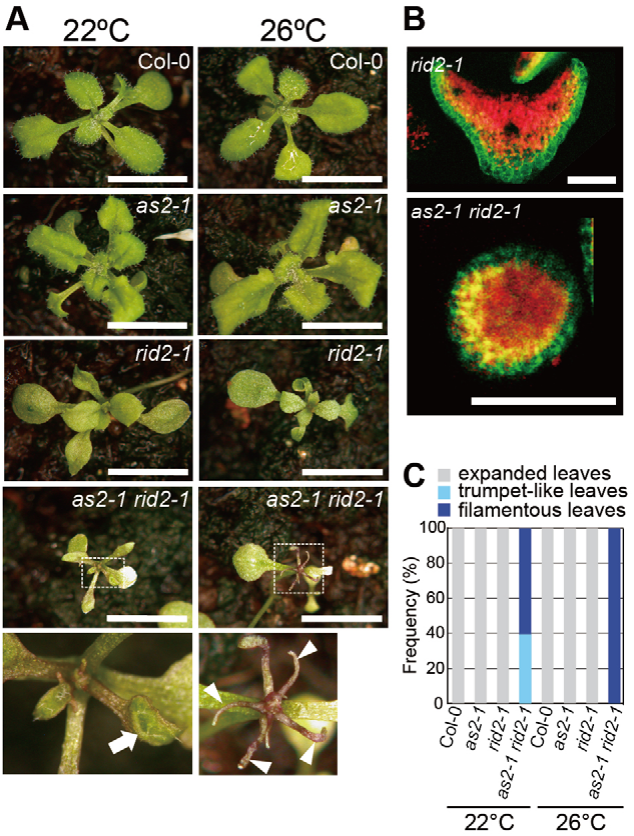
**The *rid2* mutation enhanced leaf-phenotypes in *as2-1* mutant.** (A) Gross morphologies of indicated plants grown on the soil at 22°C and 26°C and photographed at 25 days. Magnified images of the shoot apices of *as2-1 rid2-1*, which correspond to the respective boxed regions above, are shown (bottom row). The arrow and arrowheads indicate a trumpet-like leaf and filamentous leaves, respectively. Scale bars=10 mm. (B) Expression patterns of *FILp*:*GFP* in leaf primordia of *as2-1 rid2-1*, grown at 26°C. Fluorescence signals of *FILp*:*GFP* in transverse sections of leaf primordia of indicated plants grown at 26°C. Green, GFP signals; red, autofluorescence. Scale bars=100 μm. (C) Quantitative analysis of leaf polarity in Col-0 and mutant plants. Rosette leaves classified into filamentous, trumpet-like, and expanded leaves on each plant grown at 22°C and 26°C were counted at 22 days. Plants (*n*=38-106) were used for each count. Frequencies of trumpet-like or filamentous leaf were determined as described in Fig. 1D.

We introduced the temperature-sensitive mutation *rid2-1* into *as2-1* to generate the *as2-1 rid2-1* double mutants and observed phenotypes of those leaves at 22°C and 26°C. As shown in Fig. 6, the *rid2-1* single mutation produced pointed leaves and 60% of the *as2-1 rid2-1* double mutants generated filamentous leaves even at 22°C (Fig. 6C). Although *rid2-1* produced no filamentous leaves, 100% of the plants with double mutations generated short filamentous leaves at 26°C (Fig. 6A,C). Analyses of transcripts in a*s2-1 rid2-1* showed that similarly as in the *as2-1 rh10-1* double mutants (Fig. 4) transcript levels of many abaxial genes (*ETT*/*ARF3*, *ARF4*, *FIL*, *YABY5*, *KAN1*, and *KAN2*), and all four class 1 *KNOX* genes, were increased by four- to six-fold (Fig. S3B). The mutation phenotypes were suppressed by introduction of *ett-13* and *arf4-1* into *as2-1 rid2-1* (Y.Mat., C.M. and Y.Mac., unpublished data). The highly efficient generation of filamentous leaves and patterns of up-regulation of downstream genes in *as2-1 rid2-1* are reminiscent of those in *as2-1 rh10* (Figs 1 and 4).

We observed GFP signals due to FILp:GFP in the filamentous leaves generated by *as2-1 rid2-1*. The GFP signals were detected in the epidermis of the filamentous leaves (Fig. 6B); thus they were surrounded by abaxialized epidermis, suggesting that adaxial development was blocked during leaf development of *as2-1 rid2-1*.

## DISCUSSION

### Cooperative repression by AS1-AS2 and the nucleolar proteins

**Fig. 7.**
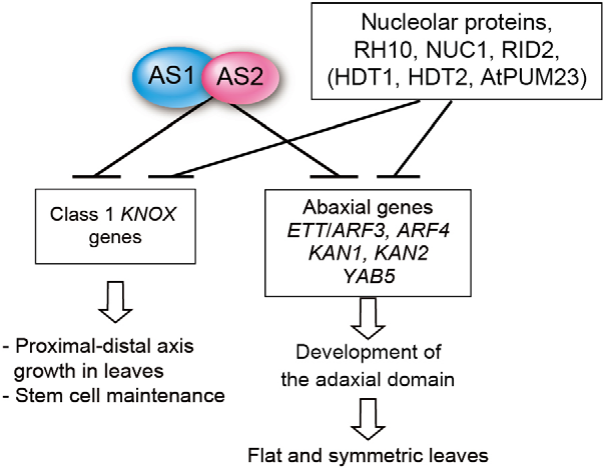
**Roles of AS1-AS2, *RH10, NUC1,* and *RID2* in leaf development in *A. thaliana***. The AS1-AS2 complex and RH10 (or NUC1, RID2) act cooperatively to repress the levels of transcripts of leaf abaxial-determinant genes, which include *ETT*/*ARF3* and *ARF4,* and of ‘meristem’ genes, namely class 1 *KNOX* genes. Repression of *ARF*s is crucial for the establishment of adaxial-abaxial polarity and then formation of flat and symmetric leaves. Class 1 *KNOX* genes are similarly repressed by AS1-AS2 together with genes for the nucleolar proteins, which is critical for proximal-distal axis growth of leaves and stem cell maintenance. See details in Discussion.

As we have reported here, the *Arabidopsis rh10-1* mutation is a novel modifier that markedly enhances the adaxial defect of the *as2* mutant to generate abaxialized filamentous leaves with the loss of the adaxial domain (Figs 1 and 2). Further molecular and genetic analyses of the *as2 rh10-1* double mutant have shown that the severe adaxial defect is attributable to synergistically increased transcript levels of abaxial genes *ETT*/*ARF3* and *ARF4* in the double mutant (Fig. 4). These results suggest the involvement of a cooperative action of nucleus-localized protein complex AS1-AS2 and nucleolus-localized protein RH10 in repression of *ETT*/*ARF3* and *ARF4* in the presumptive adaxial domain of leaf primordia, which triggers the development of the adaxial domain (Fig. 7). A similar repression mechanism also operates on the repression of all four class 1 *KNOX* genes during development of leaf primordia (Fig. 4A; Fig. 7) which are involved in proximal–distal growth, and formation of the prominent mid vein and stem cell maintenance in leaf primordia (Semiarti et al., 2001; Guo et al., 2008; Ikezaki et al., 2010). Since 54 out of 72 yeast components of the SSU processome are conserved in *Arabidopsis* (Table 1), we predicted the presence of an SSU processome-like complex, including RH10, in the plant. Taken together with results of experiments with *nuc1* and *rid2* mutants, the present results also predict that there might be a molecular link between the nucleolar complex and the AS1-AS2 complex that might cooperatively repress the abaxial genes and class 1 *KNOX* genes during leaf development. In addition, the observation that AS1 and AS2 are colocalized as a few speckles called AS2 bodies to regions adjacent to the nucleolus (Ueno et al., 2007; Luo et al., 2012a) implies a substantial link between these complexes. To approach the mechanism behind the link, molecular investigations should be further required with mutations of genes encoding core components of the SSU processome in Table 1.

It cannot be ruled out that the abnormal phenotypes observed in the present study might have also been due to a combinatorial effect of impairments unrelated to *rh10-1* that might be induced at high temperatures.

### Involvement of other nucleolar factors in the cooperative repression

With respect to nucleolar factors and leaf polarity establishment*,* we should mention that two genes encoding nucleolar proteins are also involved in rRNA biogenesis and leaf polarity establishment. Knockdown of the *Arabidopsis* genes *HDT1* and *HDT2* for nucleolar histone deacetylases (HDACs) enhances the leaf adaxial defects of *as1* and *as2* to generate the severely abaxialized filamentous leaves as seen in *rh10-1* (Ueno et al., 2007). These HDACs are responsible for establishment of nucleolar dominance, which is an epigenetic silencing of a set of rDNAs in genetic hybrids of *Arabidopsis* and is correlated with heterochromatic states of rDNAs in nucleolus organizing regions (NORs) (Pontes et al., 2007). These observations suggest that the proper silencing status of rDNAs might be critical for repression of the abaxial genes in *as1* and *as2* backgrounds. In addition, NUC2, another plant nucleolin (see Table 1), might be related to leaf phenotypes of *as2 rh10-1*, because NUC2, a negative regulator of CG methylation in rDNAs (Durut et al., 2014), is up-regulated in *nuc1*.

*Arabidopsis* nucleolar protein APUM23 with its pumilio-like RNA-binding repeats is involved in the processing of 35S pre-rRNA (Abbasi et al., 2010). The double mutants *apum23 as1* and *apum23 as2* produce filamentous leaves (Huang et al., 2014), suggesting involvement of APUM23 in leaf development.

It is worth noting that *Arabidopsis* double mutants of the ribosomal protein gene *RPS6* with *as2* exhibit the strongest adaxial defects of leaves among *as2* mutants combined with other mutations of ribosomal protein genes (Horiguchi et al., 2011). Rps6 of budding yeast is one of five ribosomal proteins (Rps4, Rps6, Rps7, Rps9, and Rps14) that were identified as bona fide components of the processome (Bernstein et al., 2004). The remaining four out of these five genes have not yet been tested for a role in leaf development. In addition, five genes for ribosomal proteins in the small subunit have been examined and three of these five exhibit adaxial defects in the *as2* background (Horiguchi et al., 2011). Seven out of ten genes for ribosomal proteins in the large subunit also exhibit the adaxial defect in the *as1* and the *as2* background (Pinon et al., 2008; Yao et al., 2008; Horiguchi et al., 2011; Szakonyi and Byrne, 2011). It would also be intriguing to investigate relationships between the adaxial defects and ribosomal protein genes for nucleolar complexes such as a large subunit (LSU) processome (McCann et al., 2015) in the nucleolus of *Arabidopsis*. It is also interesting to study relationships between AS1-AS2 and recently identified nucleolar protein AtNUFIP (Rodor et al., 2011).

### How do AS1-AS2 and RH10 cooperatively repress the downstream genes?

Although our results predict that AS1-AS2 and nucleolar proteins in the putative SSU processome-like complex might cooperatively repress the abaxial genes and class 1 *KNOX* genes, molecular mechanisms behind the cooperative action remain unelucidated. Presently, there are two classes of hypotheses to explain this cooperative repression.

#### Class 1

In general, sufficient amounts of ribosomes might provide a favourable situation to control expression of a certain group of genes involved in organ development and cell proliferation. In the wild-type plant, the cooperative repression as reported here might be explained by assuming a combinatorial effect of global enhancement of translational capacity and spatio-temporal repressive regulation of the target genes such as *ETT/ARF3*, *ARF4* and class 1 *KNOX* genes by AS1-AS2. In the *rh10* mutant, the ratio of 18SrRNA/25SrRNA is reduced (Fig. 3D) implying a decrease in ribosome amount which could cause limit the general translational ability, and which might lead to up-regulation of these target genes in the absence of AS1-AS2; however according to this model, a question still remains concerning how the maintenance of translational capacity might be involved in repression of transcript levels of these developmentally critical genes. Specific ribosomes are involved more or less efficiently in translations of specific transcripts (Nishimura et al., 2005; Wang et al., 2013; Xue and Barna, 2012). It is, however, less likely that RH10 might affect biogenesis of specific ribosomes.

#### Class 2

Recently it has been reported that the nucleolus and its peripheral regions offer a molecular architecture such as nucleolar-associated domains (NADs) (van Koningsbruggen et al., 2010; Németh et al., 2010; Padeken and Heun, 2014) that function in the regulation of gene transcription. Most rDNA loci are condensed as heterochromatin in the periphery of the nucleolus, and in the case of yeast and animal chromosomes specific sequences might also locate around the nucleolar surface, which might form distinct nuclear subcompartments (Thiry and Lafontaine, 2005; Neumüller et al., 2013). In *Arabidopsis*, DNA methyltransferase (*MET1*), nucleolar histone deacetylase (*HDA6*) and chromatin assembly (*CAF1*) genes are involved in formation of such a subcompartment (Pontvianne et al., 2013). AS2 and AS1, which is associated with HDA6 (Luo et al., 2012b), are colocalized to AS2 bodies adjacent to the nucleolus (Ueno et al., 2007; Luo et al., 2012a). Mutant proteins of AS2 that do not form AS2 bodies were not functional [L. Luo (Nagoya University, Nagoya, Japan), C.M. and Y.Mac., unpublished data]. If a plant has nuclear and nucleolar structural organizations similar to those of yeast and animals, the cooperative repression might be explained by assuming a certain physical interaction between these nucleus- and nucleolus-associated subcompartments, AS2 bodies, and the SSU processome-like complexes. Mutations in the *RH10*, *NUC1*, and *RID2* might affect integrity of nuclear subcompartments, which might alter transcriptional patterns of genes locating in and around the subcompartments. If target genes of AS1-AS2 are associated with the subcompartment, proper structural networks between the subcompartments, the nucleolar complex and the AS1-AS2 repressor might be required for cooperative repression of the target genes.

The nucleolus can also play a role in sequestration of a certain protein in the ‘detention centre’. This mechanism might affect action of proteins (positively or negatively) that are implicated in transcriptional and post transcriptional controls (Jacob et al., 2013). The sequestration is driven by long non-coding RNAs in the nucleolus, where some of these RNAs are transcribed and/or processed. It is intriguing to investigate a molecular link between the cooperative epigenetic repression by AS1-AS2 and these nucleolar events.

## MATERIALS AND METHODS

### Plant materials and growth conditions

*Arabidopsis thaliana* ecotype Col-0 (CS1092) and the mutants *as2-1* (CS3117) and *as1-1* (CS3374) were obtained from the Arabidopsis Biological Resource Center (Columbus, OH, USA; ABRC). We outcrossed *as2-1* with Col-0 three times and *as1-1* with Col-0 once, and used the progeny for our experiments (Kojima et al., 2011). Details of *ett-13* and *arf4-1* (Pekker et al., 2005), *nuc1-1* (Durut et al., 2014), *parl1-2* (SALK_002764: Petricka and Nelson, 2007), and *rid2-1* (Ohbayashi et al., 2011) were described previously. *nuc1-1* and *parl1-2* were on the Col-0 (WT) background and *rid2-1* was on the *Ler* background. For phenotypic analyses, seeds were sown on soil or on gellan gum-solidified Murashige and Skoog (MS) medium. After 2 days at 4°C in darkness, plants were transferred to a regimen of white light at 50 µmol m^−2^ s^−1^ for 16 h and darkness for 8 h daily at 16°C, 22°C, 26°C, or 28°C, as described previously (Semiarti et al., 2001). Ages of plants are given in terms of numbers of days after sowing

### Isolation of the *east2*/*rh10-1* mutant and molecular cloning of *east2*/*rh10-1*

To isolate the *east2*/*rh10-1* mutant, *as2-1* was screened as described previously (Kojima et al., 2011). The F2 population of a cross between *east2*/*rh10-1* and *Ler* was used for the genetic mapping of *east2*/*rh10-1*; the mapping primers were described previously (Kojima et al., 2011).

### Fluorescence microscopy

We crossed *as2-1 rh10-1* and several fluorescence lines, *FILp:GEP* (Watanabe and Okada, 2003), *YFP:HDT1* (Ueno et al., 2007), and *35S:Nucleolin:GFP* (Kojima et al., 2007). We introduced *FILp:GFP* into *rh10-1*, *as2-1 rh10-1*, *nuc1-1*, *as2-1 nuc1-1*, *rid2-1*, and *as2-1 rid2-1* mutants by genetic cross. Fluorescence in shoot apices was observed as described by Ishibashi et al. (2012).

### Histological analysis and observation of plant morphology

Plant tissues were prepared for thin sectioning as described by Iwakawa et al. (2007). Plant morphology was observed with a Scanning Electron Microscope XL30 (Philips Electron optics, Hillsboro, USA), and a Zeiss Stemi SV11 Apo Microscope Stereo (Carl Zeiss Inc., Oberkochen, Germany).

### Construction of *RH10-GFP*

A cDNA fragment of the *RH10* gene coding region was cloned into the binary vector pGWB6 containing the coding sequence for sGFP by using Gateway™ cloning technology (Invitrogen, Inc.) to produce binary vector-carrying genes encoding the RH10-GFP fusion protein under control of the CaMV35S promoter (RH10-GFP). RH10-GFP was transformed into plants by Agrobacterium-mediated transformation (Bechtold and Pelletier, 1998). Primers for this construction are listed in Table S6.

### Real-time RT-PCR

Leaves and shoot apices of mutant and wild-type plants were harvested at 15-days after vernalization, immediately frozen in liquid nitrogen, and stored at −80°C. Manipulations of RNA isolation and RT-PCR were performed as described by Iwakawa et al. (2007). Primer sets are listed in Table S6. The mean and standard deviation were calculated with values of more than three experimental replicates. Results were normalized by reference to the results for *ELONGATION FACTOR1a*, *TUBULIN6* and *ACTIN2* transcripts.

### Analysis of rRNA and the precursors

Preparation of total RNAs from 15-day-old plants and hybridization with the digoxigenin-labeled probe specific for ITS1 were performed as described by Ohbayashi et al. (2011). The probe was synthesized with primers ITS1 F and ITS1 R (Table S6). Gels were stained with GelRed™ (Biotium Inc., Hayward, CA, USA).

### Analysis of relative values of the 18S/25S rRNA ratio

Total RNAs were prepared from shoots excluding cotyledons of 15-day-old Col-0 and *rh10-1* plants. Levels of 18S and 25S rRNAs were measured by quantitative RT-PCR (primer listed in Table S6), and the ratio of the 18S level to the 25S level in each plant was calculated. Relative values of the *rh10-1* ratio to the Col-0 ratio were calculated.

### Microarray analysis

Dataset arrays were described previously (Takahashi et al., 2008, 2013). For Dataset-RH10-26 (Col-0, *as2-1*,* rh10-1* and *as2-1 rh10-1* grown at 26°C) and Dataset-RH10-22 (Col-0, *as2-1*,* rh10-1* and *as2-1 rh10-1* grown at 22°C), total RNA was extracted from each sample of shoot apices of 15-day-old plants and reverse transcribed, yielding double-stranded cDNA which was transcribed *in vitro* in the presence of biotin-labeled nucleotides using a GeneChip 3′ IVT Express Kit (Affymetrix Inc.). Labeled amplified antisense RNA was hybridized to Affymetrix ATH1 GeneChip arrays. Arrays were measured for fluorescence intensity with an Affymetrix GeneChip Scanner 3000 7G. The microarray data presented in this paper are available from the Gene Expression Omnibus (http://www.ncbi.nlm.nih.gov/geo/) under the accession number GSE78016.

### Data processing

Raw data processing was performed by using Affymetrix Gene Chip Operating Software (GCOS) (Version 1.4.0.036). We used two sets of gene expression data, Dataset-RH10-26 and Dataset-RH10-22. Each set of array data comprised 22,746 plant genes (probe sets). Initially, we calculated the expression signals for all strains and the log2(ratio) for three strains (*as2-1*, *rh10-1* and *as2-1 rh10-1* grown at 26°C) and three strains (*as2-1*, *rh10-1* and *as2-1 rh10-1* grown at 22°C) against Col-0 by GCOS. In this experiment, we excluded 64 controls and 2,177 genes subject to cross-hybridization, according to NetAffx Annotation (www.affymetrix.com). Furthermore, for each group set, we excluded those genes for which all strain data sets showed an absent call (i.e., a detection call determined by GCOS based on the *P*-value of the one-sided Wilcoxon 5 signed-rank test; an absent call means *P*≥0.065, which is the default threshold in GCOS), because it indicates that the expression signal was undetectable. We also excluded those genes for which all strain data sets showed a no change call [i.e., no change call means (1-0.006)≥*P*≥0.006 for one-sided Wilcoxon signed-rank test], because no change indicates that the expression signal is almost equal to that of Col-0. Thus, 4759 and 5623 genes were selected for Dataset-RH10-26 and Dataset-RH10-22, respectively. Among these data, the log2(ratio) values that were >two-fold or <0.5-fold were rounded to two-fold and 0.5-fold, respectively; the log2(ratio) values with no change were rounded to 0, to avoid category proliferation in clustering.

### Gene-list-4 for *Arabidopsis thaliana*

In the present study, we used KB-FuzzyART. This is a powerful algorithm for clustering of biological data (Takahashi et al., 2008, 2013); however, KB-FuzzyART is a knowledge-based system that requires some knowledge data. Therefore, we constructed Gene-list-1 for *A. thaliana* genes in a previous study (Takahashi et al., 2008). We added 61 genes (e.g., chromatin-related genes, ACS family, LSH family and *PIN* genes) to update Gene-list-2 (Takahashi et al., 2013), and further added 73 genes and renamed it Gene-list-4, which comprises 486 genes. Further detailed information on Gene-list-4 is shown in Table S1.

### Clustering by KB-FuzzyART

To know the effect of the *rh10-1* mutation, we applied the knowledge-based fuzzy adaptive resonance theory (KB-FuzzyART) (Takahashi et al., 2008, 2013) to the clustering of the gene expression profiles that were obtained by microarray analysis (with the Affymetrix GeneChip) of mRNAs from shoot apices of Col-0, *as2-1*, *rh10-1* and *as2-1 rh10-1* grown at 26°C for 15 days (Dataset RH10-26). The 486 genes in Gene-list-4 (Table S1) were filtered and 332 genes were excluded that have undetectable calls (Abs.), no-change calls (N.C.), or no probes on the ATH1 chip against the wild type in *as2*, *rh10-1* or *as2-1 rh10-1* plants. The expression data sets of the 154 filtered genes were processed by KB-FuzzyART and classified into 25 clusters. Then, the 22,746 probes on the GeneChip were filtered and 17,052 genes were excluded. The remaining 5694 genes were assigned either as members of each cluster or as six outliers. The patterns of gene expression levels in the outliers did not match those in any clusters. The outliers comprised 229 of the 5694 genes. Profiles of the constructed clusters and gene assignments are shown in Tables S2 and S3. We sought for genes whose levels of expression were increased in the *as2-1 rh10-1* double mutant when compared with those in the wild type grown at 26°C, as shown in Table S2. Expression levels of genes in Clusters h1 were upregulated in the *as2 rh10-1* double mutant. Clusters h1 included *KNAT2*, *KNAT6*, *STM*, *KAN2*, *KRP2*, *KRP5* and *IPT3*, which have also been identified downstream of modifier genes, such as *EAL1* and *ELO3* (Takahashi et al. 2013). Clusters h14 included *BP/KNAT1* and Clusters h17 included *ETT/ARF3* and *YAB5* (Table S3, Fig. S2A).

We also applied KB-FuzzyART to the clustering of gene expression profiles that were obtained by microarray analysis (with the Affymetrix GeneChip) of mRNAs from Col-0, *as2-1*,* rh10-1* and *as2-1 rh10-1* grown at 22°C for 15 days (Dataset-RH10-22). The expression data sets of the 129 filtered genes were processed by KB-FuzzyART and classified into 19 clusters. Then, the 22,746 probes on the GeneChip were filtered and 17,709 genes were excluded. The remaining 5037 genes were assigned either as members of each cluster or as outliers (Table S4). The outliers comprised 184 out of 5037 genes. The clusters that have patterns of increased transcript levels in the *as2-1 rh10-1* double mutant did not include abaxial determinant genes and class 1 *KNOX* genes, except for *YAB5*, as shown in Table S5.

### *In silico* homology analyses to identify *Arabidopsis* orthologs of proteins in the SSU processome

We obtained amino acid sequences of 72 yeast proteins and 66 human proteins in the SSU processomes from NCBI RefSeq (www.ncbi.nlm.nih.gov/refseq), using gene names and accession numbers of the proteins listed by Phipps et al. (2011). *Arabidopsis* proteins that were homologous in terms of amino acid sequences to these yeast, and human proteins were obtained from Ensembl Release 83 (www.ensembl.org). Homology searches were conducted by BLAST Ver. 2.2.25 separately for these yeast and human sequences. Total 1157 proteins representing 482 genes of *A. thaliana* were extracted by BLASTp (*E*<e^−10^) from the *Arabidopsis* database commonly on the basis of these yeast and human sequences (Table S7). The bootstrapping phylogenetic tree analyses were conducted by ClustalW Ver. 2.1 with default parameters by using the website of the DNA Data Bank of Japan (DDBJ). For our phylogenetic tree analyses, we selected one by one the branch of the tree that was the closest to each protein in yeast and/or human SSU processomes to identify *Arabidopsis* orthologs.
